# Research progress on the GRP78 gene in the diagnosis, treatment and immunity of cervical cancer

**DOI:** 10.1186/s40001-023-01241-0

**Published:** 2023-10-20

**Authors:** Yingying Bai, Wenhua Wang, Yuemei Cheng, Yongxiu Yang

**Affiliations:** 1 Department of Gynecology and obstetrics, Tangdu Hospital, Air Force Medical University, 569Xinsi Road, Baqiao District, Xian, 710038 China; 2 The First Clinical Medical College of Lanzhou University, Lanzhou, Gansu, People’s Republic of China; 3 Department of Obstetrics and Gynecology, First Hospital of Lanzhou University, Lanzhou, Gansu People’s Republic of China; 4 No.1, Dong gang West Road, Cheng guan District, Lanzhou, Gansu People’s Republic of China

**Keywords:** GRP78 gene, ER, Cervical cancer, Chemoresistance, Immune application, Diagnostic markers

## Abstract

**Background:**

GRP78 is a molecular chaperone protein in the endoplasmic reticulum that is involved in protein assembly and quality control, and it participates in ER stress regulation of endoplasmic reticulum stress pathways. Studies have confirmed that GRP78 gene is highly expressed in a variety of tumors and is involved in different biological functions.

**Purpose:**

The present review highlights the involvement of the GRP78 gene in regulating the development of cervical cancer by promoting the proliferation and invasion of cervical cancer cells as well as by inhibiting apoptosis and promoting the Warburg effect. High expression of GRP78 is positively correlated with chemotherapy resistance in cervical cancer. GRP78 plays an anticancer role in cervical cancer by regulating autophagy and apoptosis. Mediated immune CD8 + T cells regulate tumor cell immunity and play a role in the application of the HPV vaccine.

**Conclusions:**

GRP78 plays a multifunctional role in cervical cancer and has important therapeutic and diagnostic value.

## Introduction

Cervical cancer is a growing global burden, especially in developing and industrialized countries. In 2020, there were 604,127 new cases of cervical cancer and 341,831 deaths worldwide [1]. The mortality rate of cervical cancer in transition countries is significantly higher than that in developing countries. However, in middle-income countries, cervical cancer is the second most common type of cancer and the third most common cause of cancer death [2]. In china, 2022 statistics showed that the number of new cases of cervical cancer has reached 109,700 per year, and the annual death rate has reached 59,100, which seriously endangers women’s health. In particular, advanced, recurrent and metastatic cervical cancer has a poor prognosis, with a survival rate of only 17%.

At present, surgery is the main treatment for early cervical cancer [3]. According to the postoperative pathological risk factors, whether to combine postoperative radiotherapy and chemotherapy is considered. For Locally advanced cervical cancer (LACC), simultaneous chemoradiotherapy is the standard treatment mode. For stage IIB and above or advanced distant metastatic cervical cancer, the NCCN guidelines recommend chemoradiation depending on the patient's situation. For those who cannot tolerate surgery, radiotherapy is the best treatment, but there are high risk factors after radical hysterectomy, radiotherapy can be selected as adjuvant therapy. Despite surgery, radiation and chemotherapy, the recurrence rate is 35%. The survival rate of cervical cancer patients largely depends on early diagnosis and intervention. A better understanding of the molecular mechanisms involved in the initiation and progression of cervical cancer as well as the development of drug resistance and therapeutic interventions will help to discover new therapeutic strategies [4]. With the continuous in-depth research of tumor targeted therapy, targeted therapy for cervical cancer has been widely recognized clinically. At present, specific molecular targeted drugs against tumors have been gradually identified, such as the anti-angiogenic drug Bevacizumab and VEGFR receptor inhibitor Apatinib are combined with chemotherapy drugs to treat advanced cervical cancer, and have achieved good therapeutic effects [5–8].

In recent years, there has been continuous research on endoplasmic reticulum stress- and GRP78-regulated unfolded protein response (UPR) in cancer cells. GPRP78 gene database showed that significantly high expression was reported in highly malignant and aggressive glioblastoma, lung cancer, breast cancer, colon cancer, and liver cancer [9–12]. These data confirm that GRP78 can be present in malignant cells and endothelial cells, but is rarely expressed in normal cells and is involved in tumor cell metastasis and tumor drug resistance, suggesting that GRP78 is a promising cancer-specific biomarker and a target for cancer imaging and therapy [13, 14]. New research has found that GRP78 is involved in the regulation of the biological behavior of cervical cancer, and it has a certain correlation with the prognosis, drug resistance and immune invasion of cervical cancer [15]. Tumor stem cells in tumor maintain the vitality of tumor cell population through self-renewal and infinite proliferation. Yan Zhong et al. found that EIF3D promoted the activation of FAK in cervical cancer cells through GRP78, maintaining the characteristics of cervical cancer stem cells and thus promoting the progression of cervical cancer [16]. However, the regulatory mechanism between GRP78 and cervical cancer is not clear.

**Fig. 1:**
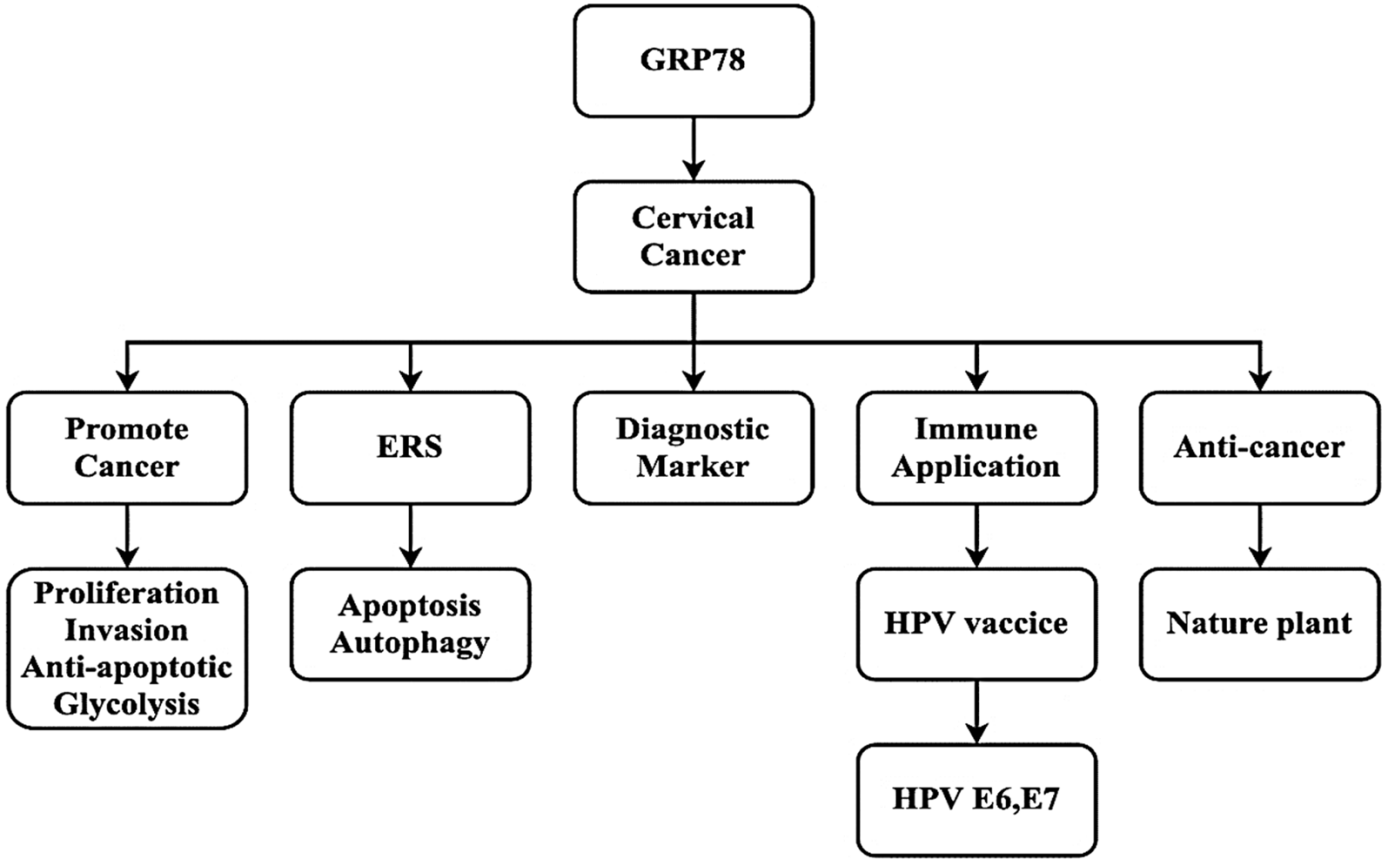
Multifunctional display of GRP78 in cervical cancer

In this paper, the mechanism of GRP78 involved in the regulation of cervical cancer behavior is reviewed, and the current application of GRP78 in the treatment, diagnosis and immunology of cervical cancer is discussed. The function of GRP78 gene involved in cervical cancer is summarized, and the exploration of GRP78 in cervical cancer is promoted. GRP78 gene is expected to become a potential new target for cervical cancer diagnosis and treatment, and may eventually improve the prognosis of patients with cervical cancer. The flow chart of this study is shown in Fig. 1.

## Endoplasmic reticulum

The endoplasmic reticulum (ER) is the largest compartment in eukaryotic cells. It has been reported that ER plays an important role in antibody and hormone secretion, protein folding and protein quality control, among which molecular chaperone plays an important role in protein quality control and protein folding [9, 17, 18], glucose-regulatory protein (GRP) is a key chaperone in ER, when misfolded proteins accumulate, GRP78/BiP and ER effector molecules IRE1α, PERK, and XBP1s are activated, thus initiating the UPR response [19]. A mild UPR response can protect cells, and if ER stress is high or persistent, UPR can regulate cell death through different pathways. Thus, ER mediated GRP78 and URP responses are associated with cell death, tumor development and drug resistance, and also play an important role in tumor concomitancy.

## Functions of GRP78 protein

Glucose-regulating protein 78 (GRP78) is an executor of the unfolded response in the endoplasmic reticulum lumen and a chaperone in the heat shock protein family 70 that regulates cellular responses to unfolded and misfolded proteins [20, 21]. GRP78 is encoded by the human HSPA5 gene and is an ER homolog of HSP70 with 60% amino acid sequence similarity, and it consists of two major functional domains, namely, an N-terminal nucleotide binding domain (NBD) with ATPase activity and a C-terminal substrate binding domain (SBD) [22]. Nucleotide-binding domain (NBD), which binds ATP, plays a crucial role in promoting the correct folding of proteins and is conducive to cell survival under stress, while substrate binding domain (SBD), nucleotide-binding domain (NBD), Ability to bind polypeptides/proteins [23, 24]. GRP78 and NBD and substrate binding domain (SBD)) bind to promote the initiation of endoplasmic reticulum associated protein degradation ERAD, when the unfolded/misfolded proteins in the ER exceed the capacity of the protein folding mechanism, which initiates the unfolded protein response UPR [23].

**Fig. 2:**
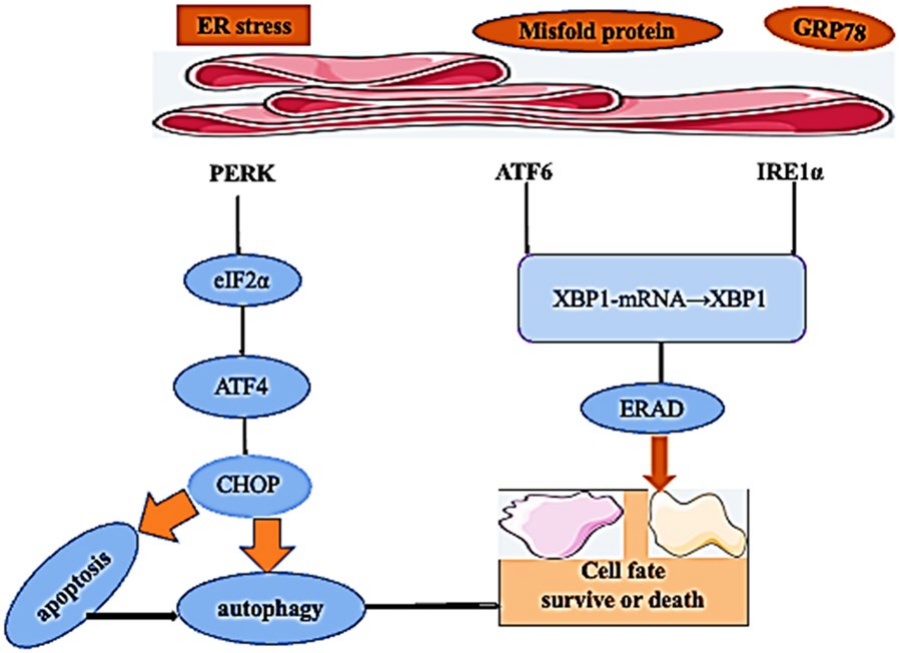
GRP78 contains the KDEL ER localization signal, and it exists in the ER in its intracellular form. GRP78 is a key participant in the unfolded protein response (UPR), and its ATP-binding domain plays a crucial role in promoting the correct folding of proteins, which is beneficial to cell survival under stress conditions. When ER stress occurs continuously, GRP78 activates PERK, IREα and ATF6, which induces apoptotic inflammation, leading to cell death

As a resident protein of the ER, GRP78 functions as a molecular chaperone that binds to misfolded proteins and unassembled complexes, and it initiates ER-associated degradation responsible (ERAD) for UPR regulation. GRP78 binds to unfolded peptides via a SBD and gains energy through ATP hydrolysis via the NBD to prevent aggregation. GRP78 binds in an inactive form to activated transcription factor 6 (ATF6), protein kinase RNA-like endoplasmic reticulum kinase (PERK) and inositol kinase-1 (IRE1) under standard conditions of cellular homeostasis (homeostasis), and it is a transmembrane stress sensor for UPR. When cells are exposed to accumulated unfolded proteins in the ER, the release of GRP78 from the UPR sensor activates the UPR [25], and the sustained UPR stimulates the cell death pathway (Fig. 2).

GRP78 has traditionally been considered an endoplasmic regulating cavity protein. However, there is increasing evidence that GRP78 is also detected at other cellular sites, including the cell surface, cytoplasm, mitochondria and nucleus, and it participates in signal transduction, proliferation, invasion, apoptosis, inflammation and immunity [26] Importantly, one of the branches of GRP78 is redirected to the surface of specific types of cells, such as cancer cells, and this process is actively enhanced by endoplasmic reticulum stress to regulate different biological functions of cancer [14, 27, 28].

## Biological function of GRP78 in cervical cancer

**Fig. 3:**
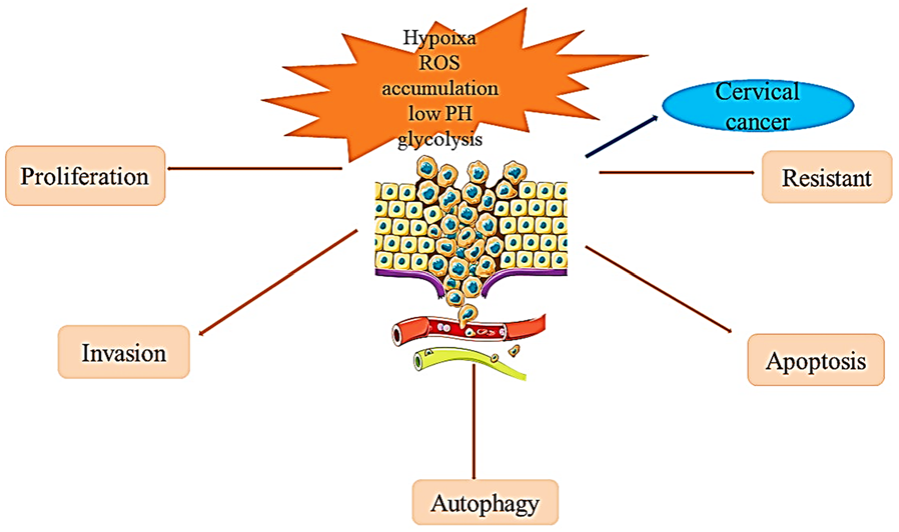
Function of GRP78 in cervical cancer. The complex tumor environment in cervical cancer leads to up-regulation of GRP78 expression. GRP78 regulates proliferation, apoptosis, autophagy, invasion and chemotherapy resistance of cervical cancer cells

GRP78, as a chaperone protein in the endoplasmic reticulum, is involved in the main regulation of UPR and is highly expressed in a variety of tumor cells. Previous studies have reported that GRP78 is involved in multiple aspects of tumor biology, such as proliferation, apoptosis, autophagy [29], invasion and metastasis, and chemotherapy resistance through UPP-dependent and non-UPR-regulated functions [30] (Fig. 3). In this section, we review in detail the mechanisms of apoptosis, autophagy, metastasis and drug resistance of GRP78 in cervical cancer.

### GRP78 induces drug resistance in cervical cancer

**Fig. 4:**
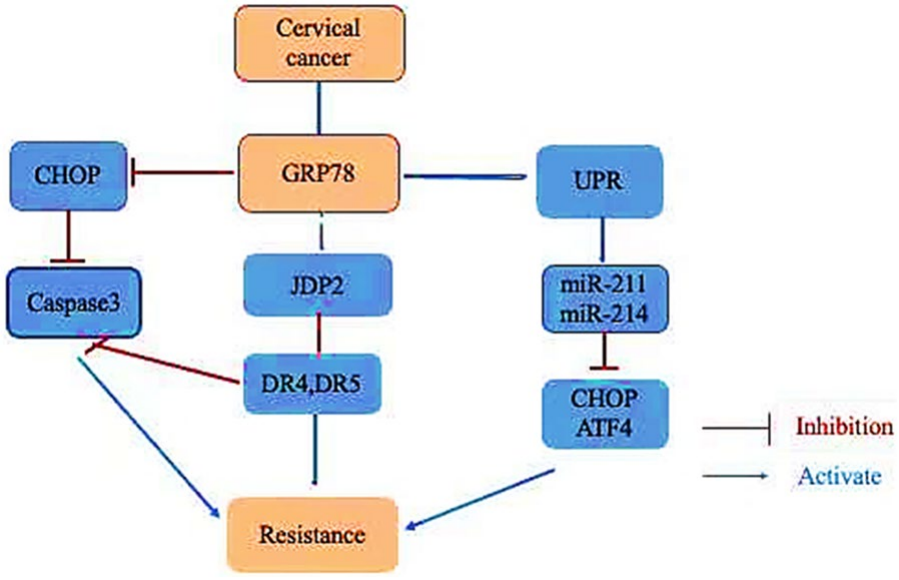
GRP78 activated UPR, up-regulated miR-214 and miR-211, inhibited the expressions of CHOP and ATF4, and inhibited the expression of apoptotic genes. GRP78, activated ATF4, and ATF4 further activated JDP2, inhibiting the expression of DR4 and DR5, inhibit cell apoptosis

Defects in apoptosis are one of the mechanisms of drug resistance in tumor cells [20]. The ultimate goal of most anticancer drugs is to induce apoptosis in tumor cells, which leads to cell death, and the destruction of the apoptosis mechanism may affect resistance to anticancer drugs. Recent studies have shown that GRP78 plays an important role in the process of tumor resistance [31, 32]. The mechanism is that GRP78 is involved in the resistance of tumor cells to chemotherapy drugs through the resistance to apoptosis [32] (Fig. 4).

In terms of chemotherapy, a variety of anticancer drugs can induce resistance of GRP78 to cancer chemotherapy drugs and promote tumorigenesis [33–38]. Research has shown that GRP78 is involved in the drug resistance of several tumor chemotherapy drugs [25]. In cervical cancer, western blot analysis has confirmed that the expression of GRP78 in cervical cancer tissues and cells is higher than that in neighboring tissues. Cervical cancer cells have the highest resistance to cisplatin after treatment with different concentrations of cisplatin. Silencing GRP78 followed by treatment with cisplatin causes more than 80% of cervical cancer cells to undergo apoptosis with upregulated caspase 3 and CHOP expression as well as downregulated BcL-2 expression [39].

Cancer cells reestablish endoplasmic reticulum homeostasis through UPR, which contributes to cell survival and leads to drug resistance. The regulation between UPR signal and microRNAs (miRNAs) is involved in the development and drug resistance of cervical cancer. It was found that miR-211 contains a sequence targeting CHOP promoter region, which inhibits the expression of CHOP and thus inhibits the pro-apoptotic signaling pathway, contributing to cell survival and the occurrence of drug resistance. miR-214 can reduce the expression of ATF4, thereby increasing the transcription of pro-survival genes and promoting the occurrence of drug resistance [40–42]. JDP 2 is a transcription factor that plays an important role in cell differentiation. Studies in HeLa cells have confirmed that the activated ER stress, the increased expression of GRP78, activated ATF4, and ATF4 further activated JDP2, thus inhibiting the expression of DR4 and DR5, inhibiting cell apoptosis, and reducing the sensitivity to TRAIL chemotherapy drugs [43].

### GRP78 promotes the development of cervical cancer (proliferation, invasion, anti-apoptosis and Warburg effect)

**Fig. 5:**
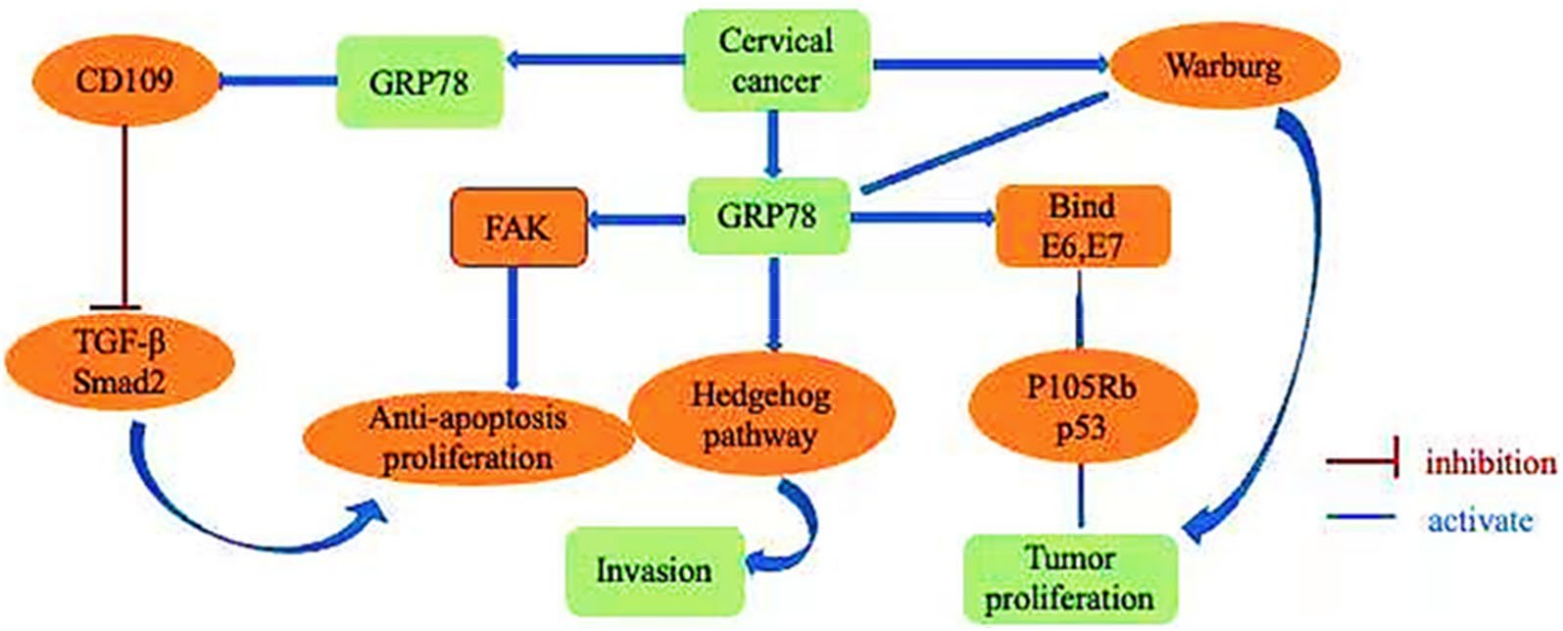
GRP78 promotes the proliferation and metastasis of cervical cancer through theTGF-β, FAK and Hedgehog pathways, and it regulates the stability of HPV E6 and 7 proteins to promote the progression of cervical cancer

Recent studies have demonstrated that the unfolded protein response (UPR) triggered by GRP78 contributes to cell transformation, the inflammatory response, tumor angiogenesis, tumor cell invasiveness and tumor cell metastasis, increasing our understanding of the relationship between ER stress and cancer biology [44]. Proteins in the ER have been identified as regulators of cancer development. Among them, GRP78 has been reported and confirmed in several studies as an independent biomarker involved in the regulation of tumor development (Fig. 5).

Researchers have investigated how GRP78 promotes tumor development. Studies have confirmed that GRP78 is involved in a variety of cellular biological processes, including translocation of newly synthesized peptides across the ER membrane, promotion of proteasomal degradation of misfolded proteins, maintenance of intracellular calcium homeostasis and function as an ER baroreceptor [45, 46]. However, it has been confirmed in cancer biology studies that stress induction of GRP78 is an important survival mechanism, and it has anti-apoptotic properties in the unfolded protein response. Upregulation of GRP78 in cancer cells is associated with increased tumor growth, invasion and metastasis. It has been confirmed that activation of FAK signaling pathway promotes the progression of cervical cancer. GRP78 is highly expressed in cervical cancer and is involved in tumor proliferation and metastasis of cervical cancer by regulating the FAK pathway [47], and downregulation of GRP78 inhibits the development of cervical cancer. An experimental cohort study has demonstrated that upregulation of GRP78 promotes tumor growth and chemotherapy resistance in subcutaneous tumor models as well as poor survival in patients. The Hedgehog signaling pathway is involved in the GRP78-mediated regulation of the proliferation and metastasis of tumor cells. In cervical cancer cells, GRP78 promotes the proliferation of cervical cancer cells through regulation of the Hedgehog pathway, thus promoting the progression of cervical cancer [48].

Defects in apoptosis are an important cause of tumor development. A previous study has found that GRP78 has an anti-apoptotic function. GRP78 knockout in HeLa cells activates the UPR pathway and increases the expression of CHOP, ERdj4 and P5 proteins, ultimately leading to cell apoptosis.

Human papillomavirus (HPV) is the main promoter of cervical cancer. The E6 protein is a nonstructural protein of HPV and is responsible for cell differentiation by targeting the tumor suppressor genes, p105Rb and p53. Studies have confirmed that the E6 protein is stabilized by the GRP78 protein to promote the development of cervical cancer. GRP78 knockout destroys the stability of E6, thus leading to faster degradation of E6 in vivo and inhibiting the development of cervical cancer [49]. The transcripts of the human papillomavirus 16 (HPV16) E6 and E7 oncogenes undergo selective RNA splicing to produce multiple splicing homologous types. The E6 and E7 proteins bind to GRP78 proteins and interact with each other, which leads to the stabilization of E6 and E7 by prolonging the half-life of each protein, thus promoting the development of cervical cancer [50].

Cancer cells use a large amount of nutrients to maintain unlimited proliferation and growth. This requires a reprogramming of energy metabolism, which is considered one of the hallmarks of cancer [51]. In the process of glycolysis, cells decompose glucose to produce pyruvate and a small amount of ATP, showing high glycolytic activity in tumor cells, and generate lactic acid by activating lactate dehydrogenase (LDH) and inhibiting the metabolism of pyruvate in mitochondria [52], which is called the Warburg effect [53].

GRP78 induces cancer cell proliferation and migration by disrupting the normal cell apoptosis process through abnormal glucose metabolism behavior [54]. Studies have shown that high expression of the EIF3D gene promotes cell growth and the Warburg effect in cervical cancer models in vitro, while inhibition of GRP78 reduces the influence of EIF3D on the Warburg effect of cervical cancer in vitro. These results confirm that GRP78 and EIF3D interact with each other to regulate the Warburg effect in cervical cancer and promote the development of cervical cancer [55]. Studies in cervical cancer have also shown that eukaryotic initiation factor (EIF3D) promotes the development of cervical cancer by activating the GRP78/FAK signaling pathway [16].

### Inhibition of cervical cancer through GRP78

It is currently believed that tumor cells interfere with the protein folding process of the endoplasmic reticulum (ER) under stress conditions, leading to ER stress (ERS). ERS can activate the UPR of cells [56]. During ER stress, when misfolded proteins accumulate in the ER, GRP78 binds to the misfolded protein, activating three effector proteins, IRE1, PERK, and ATF6, to initiate the UPR signaling cascade. Activated IRE1 cuts the unspliced XBP1 mRNA and then translates the cut mRNA into the transcription factor XBP1.PERK dimerization, trans-autophosphorylation, and then phosphorylation of eIF2α to inhibit protein translation. Phosphorylated eIF2α also activates the transcription factor ATF4. ATF6 is transferred from the endoplasmic reticulum to the Golgi complex. In the Golgi, ATF6 is cleaved by the SiP1 and SiP2 proteases, releasing the cytoplasmic alkaline leucine zipper (bZIP) domain, which is transferred to the nucleus. ATF4, XBP1s and ATF6 transcription factors synergistically control the expression of ERAD, er-chaperone, pro-apoptotic CHOP and autophagy-related adaptive genes, thereby regulating the occurrence of apoptosis and autophagy [57, 58].

#### Induction apoptosis

**Fig. 6:**
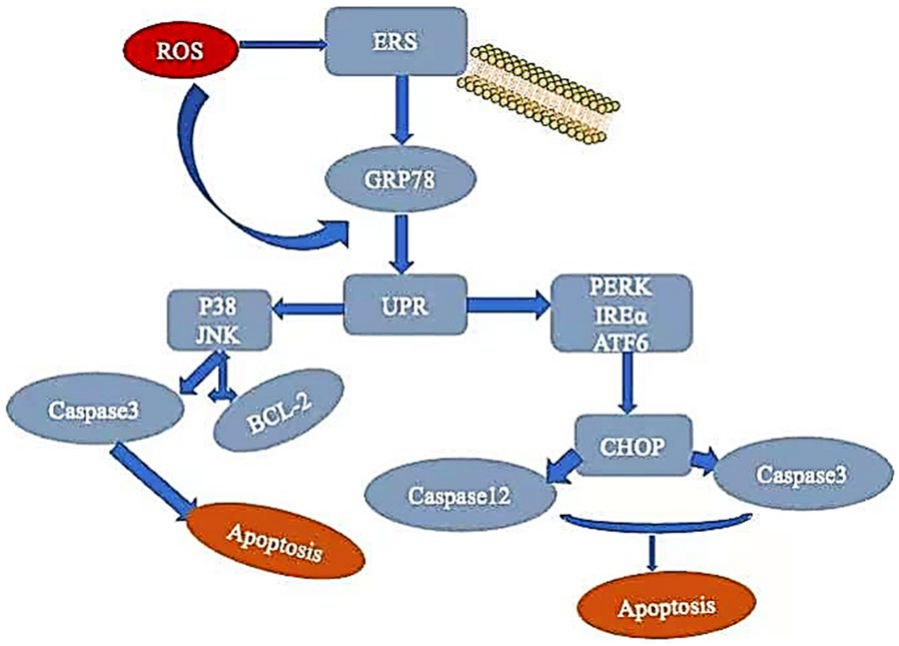
When endoplasmic reticulum stress occurs, GRP78, UPR, PERK, iREα and ATF6 increase, which activates CHOP, caspase-3 and caspase-12, thereby inhibiting the expression of Bcl-2 and inducing apoptosis. GRP78 activates the JNK and P38 signaling pathways as well as induces apoptosis

Apoptosis is an actively regulated type of programmed cell death that plays an important role in antitumor research. In recent years, several studies have evaluated the role of GRP78 in apoptosis. This is shown in (Fig. 6). Meng Wang Some scholars have confirmed that by targeting GRP78 to induce cervical cancer cell apoptosis, promote the anti-cancer effect of chemotherapy drugs, thus playing a role in the clinical treatment of GRP78 cancer inhibition [59].

Under intense and sustained ER stress, GRP78 is activated, which induces the expression of downstream CHOP, thereby inducing cell apoptosis. The accumulation of calcium ions in the endoplasmic reticulum promotes the upregulation of the Bax/BcL-2 ratio and activates the mitochondrial pathway to induce apoptosis. In addition, Ca2 + released into the cytoplasm also activates calpain, which promotes the cleavage of Caspase-12 and mediates apoptosis. JNK is also activated by dimerized IRE1 to induce apoptosis through TRAF2–ASK1 signaling [60]. Sustained ER stress leads to overproduction of ROS, while high ROS levels induce ER stress and apoptosis [61, 62].

A previous study has confirmed that quercetin has the following effects: induces endoplasmic reticulum stress in cervical cancer HeLa cells; induces the expression of GRP78; activates the increased expression of caspase-3, CHOP, IRE1, p-PERK and c-ATF6; reduces the expression of Bcl-2; induces cell apoptosis; and inhibits the viability of cervical cancer cells [63]. It has been previous reported that ultrasound therapy significantly inhibits the proliferation and induces apoptosis of cervical cancer HeLa cells by activating endoplasmic reticulum stress (ERS), which is related to the apoptosis signaling pathway, as well as by triggering JNK phosphorylation and increasing the expression of GRP78 and caspase-12 [64]. Sesamin activates GRP78/PERK/JNK to induce apoptosis of cervical cancer cells, thus significantly improving the viability of tumor cells [65]. Bortezomib activates endoplasmic reticulum stress (ERS) in cervical cancer HeLa cells and promotes the expression of GRP78, ATF4 and CCAAT, and it induces autophagy and apoptosis in cervical cancer cells, thereby inhibiting cell proliferation, resulting in anticancer activity [66]. Studies have confirmed that Protodioscin (PD) induces the up-regulation of GRP78 protein by activating ROS in HeLa cells. Downstream apoptotic proteins caspase-8, -3, -9, PARP, and Bax activation, and down-regulation of Bcl-2 expression induce apoptosis. After treating cells with ROS inhibitors, The down-regulation of GRP78 and apoptosis-related proteins suggested that ROS/GRP78/Bax pathway was involved in apoptosis induction in HeLa cells by Protodioscin (PD) and P38 and JNK pathways are activated at the same time [67]. Previous studies have shown that vetiporfen (VP) activates ER stress and induces apoptosis of HeLa and SiHa cells by upregulating the expression of GRP78, CHOP and Caspase-12, thereby inhibiting the growth of cervical cancer xenografts in nude mice [59]. Dezocine, a dual agonist and antagonist of the MUA–opioid receptor and KAPpa–opioid receptor, is widely used as an analgesic. Studies have shown that dezocine exerts anticancer activity in HeLa cervical cancer cells via upregulating the expression of GRP78, IRE1 and p-JNK to activate ER stress and induce apoptosis. When the ER stress pathway is blocked, dezocine-induced apoptosis is weakened [68].

#### Autophagy induction

**Fig. 7:**
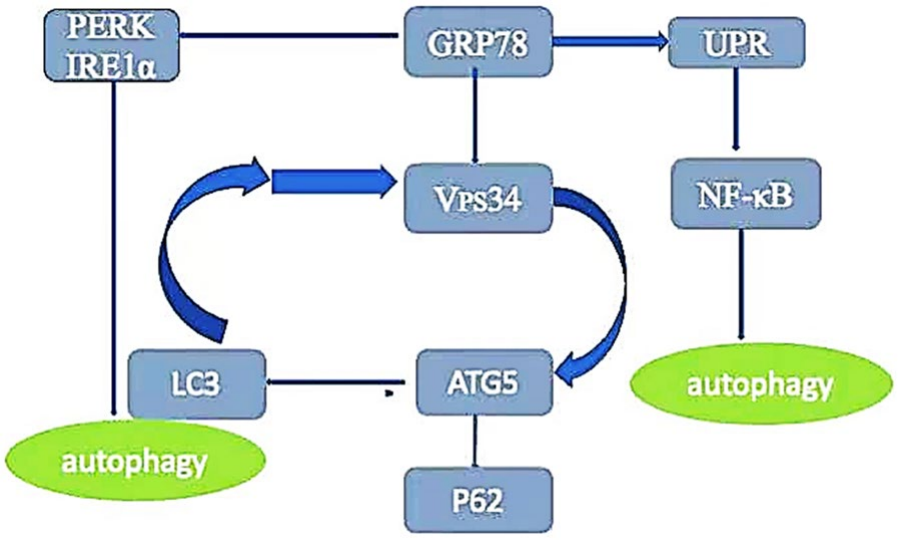
GRP78 activates the UPR and regulates PERK, NF-KB to activate LC3 and trigger autophagy. GRP78 regulates Vps34, induces the expression of ATG5 and LC3, down-regulates the expression of P62, and induces autophagy in cervical cancer cells

Autophagy plays a dual role in tumor species, but the mechanism by which autophagy determines cell death lies in the regulation of tumor suppressor genes and oncogenes in cells [69]. Beclin1 plays an important role in autophagy. Beclin1 induces autophagy and promotes cell death. The mechanism is that Beclin1 induces the activation of PI3K/Akt pathway in HeLa cells, and then the expression of apoptosis factors Bax and caspase-3, which leads to the inhibition of cell proliferation and thus cell death [70]. An increasing number of studies have shown that endoplasmic reticulum stress-induced autophagy activation promotes the death of cervical cancer cells [71] (Fig. 7).

Brefeldin is a lactone antibiotic that induces UPR activation in cervical cancer cells through endoplasmic reticulum stress, with increased IRE1α and GRP-78 protein levels and ER swelling. At the same time, endoplasmic reticular stress induced LC3 expression through activation of NF-κB and induced autophagy of cervical cancer cells. NF-κB pathway inhibitor QNZ could reverse Brefeldin-induced autophagy and reduce the death of cervical cancer cells, suggesting that GRP78/NF-κB/LC3 was involved in the autophagy death of cervical cancer cells [72] Propofol (2,6-diisopropyl phenol) is widely used in anesthesia. Studies have confirmed that propofol disrupts the intracellular Ca2 + balance by activating the AMPK/mTOR signaling pathway and endoplasmic reticulum (ER) stress, thereby enhancing autophagosome accumulation and inducing autophagic death of cervical cancer cells [73].

Recent studies have found that AGG-mediated Akt dephosphorylation leads to ER stress, inducing autophagy-dependent cell death in cervical cancer cells, and ER inhibitors reverse AGG-induced autophagic death [74]. Tobacco mosaic virus is an RNA virus. Recent data have suggested that TMV–RNA only infects plants, especially tobacco plants. Studies have found that tobacco mosaic virus (TMV–RNA) is translated into coat protein (CP) in the endoplasmic reticulum after infection of HeLa cells, and TMV-positive RNA is transferred from the cytoplasm to the nucleolus. In addition, the expression of GRP78 (a glucose-regulated protein), a typical marker of ER (ER stress), is significantly increased, and the formation of autophagosomes is closely related to the expanded ER membrane. Data have shown that HeLa cells defend against TMV–RNA virus infection through ERS and ERS-induced autophagy [75]. Vps34, a type III phosphatidylinositol kinase, is a key protein in the process of autophagy. Studies have shown that GRP78 induces the expression of LC3 and ATG5 by Vps34 activation, ultimately promoting autophagy in HeLa cells [76].

### GRP78 as a diagnostic marker in cervical cancer

In recent years, the clinical value of serum tumor markers in the early diagnosis, efficacy evaluation, prognosis and follow-up of cervical cancer has become increasingly prominent, providing new ideas for the clinical improvement of the diagnosis and treatment of cervical cancer. The development of precancerous lesions to cervical cancer is a relatively long process. Because the early stage does not have specific symptoms, many patients are diagnosed at the late stage of the cancer. Therefore, cervical cancer diagnostic markers are valuable in the diagnosis of cervical cancer.

As a member of the Hsp70 family, overexpression of GRP78 in cancer cells and human tumors leads to malignant tumors and increases the survival of cancer cells due to drug resistance [77, 78]. High expression of GRP78 is expected to become a tumor diagnostic marker. Scholars from the University of California (UCSC) have extracted the mRNA expression profiles of 306 tumor tissues and 13 normal tissues from a database and found that GRP78 is more highly expressed in cervical cancer tissues than in the normal tissues; after verification of the cervical cancer tissue samples and normal tissue samples, these researchers confirmed that patients with high GRP78 expression have poor OS [79]. In a previous study, researchers screened out differentially expressed lncRNAs in cervical cancer related to glucose-regulated protein 78 (GRP78) from a database; these researchers demonstrated that LINC00294 is positively correlated with GRP78 and that knock down of GRP78 in HeLA cells significantly downregulates LINC00294, which inhibits the Hedgehog pathway and regulates cell cycle arrest, thereby suggesting that LINC00294 may be a diagnostic target for cervical cancer [44].

### Application of an ER stress inducer involving GRP78 in immunotherapy

Immune checkpoint inhibitors and other therapies have shown powerful therapeutic effects in the treatment of a variety of cancers. Other immunotherapies have gradually attracted more attention in cancer treatment [80]. Immunotherapy induces targeting of malignant cells through the use of immune stimulation strategies, such as vaccine therapy expressing tumor-associated antigens and immunogenic cell death.

**Fig. 8:**
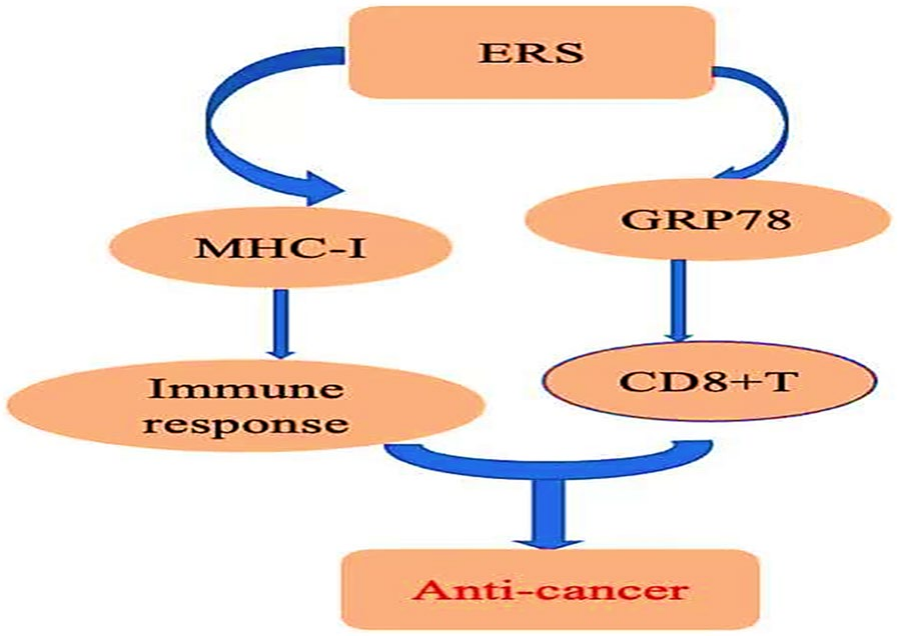
ERS plays an antitumor role by regulating tumor immunity via MHC-1 and activating CD8 + T cells

Studies have confirmed that assembly and presentation of Major histocompatibility Complex-I (MHC-I) epitopes occur in the endoplasmic reticulum [81] (Fig. 8). Therefore, the endoplasmic reticulum is important for inducing specific immune responses. At the same time, studies have confirmed that the endoplasmic reticulum enhances anti-tumor immune response by directly loading MHC-I into tumor organs [82]. It has also been found that targeting HPV-16 antigens such as E6 and E7 to the endoplasmic reticulum (ER) enhances anti-tumor immune responses [83, 84].

In cancer, ER stress activates cells of the adaptive immune system [68]. ER stress induces systemic inflammation through proteolytic activation of the cyclic AMP response element-binding protein H (CREBH) transcription factor on the ER membrane [85]. ER stress-mediated calreticulin (CRT) cell surface presentation has emerged as a damage-related molecular pattern (DAMP) of potential importance in cancer [86]. Recent results have found that the treatment of TC-1 cervical cancer tumor-bearing mice with the ER stress inducer, 3-BrPA, induces stress in tumor-bearing cells, which is accompanied by GRP78 upregulation, and produces potentially effective immune-mediated therapeutic antitumor effects by increasing the sensitivity of tumor cells to antigen-specific CD8 + T-cell-mediated killing. Therefore, the experimental results confirmed the role of the ER stress inducer, 3-BrPA, in cervical cancer immunotherapy. InDNA vaccine model experiment, it was found that fusion with hpv16e6 and E7 antigens using signals (SP and/or KDEL) activated IFN-γ and anti-tumor responses, indicating that ER induction is involved in immunotherapy response [87, 88].

### Natural products as inducers of GRP78 in cervical cancer chemoprevention

In recent years, tumor treatment mechanisms based on ER stress have attracted extensive attention [89]. When misfolded proteins accumulate in the endoplasmic reticulum lumen, an unfolded protein response (UPR) is triggered, and the regulation of the UPR can determine cell death or survival. Previous studies have confirmed that a sustained UPR regulates cell death through the PI3K/Akt/mTOR pathway or Ras/Raf/MEK/ERK pathway [90]. As shown in Table 1, a number of natural compounds with anticancer properties have been shown to promote the expression or activity of GRP78 and inhibit cervical cancer. However, in most cases, the precise molecular target has not been identified.

**Table 1:** Natural products as inducer of GRP78 in cervical cancer cells

Nature products	Pharmacological effect	Cervical cells
Dicerandrol from endophytic fungus, Phomopsis sp. Hesperidin from citrus fruits	G2/Mell cycle arrest Induce apoptosis induce ROS G0/G1cycle arrest cyclinD1 cyclinE1 inhibit	HeLa cells [91]
	Activate Caspase-3 induce apoptosis	HeLa cells [92]
Sesamin from sesamum indicun	Induce apoptosis Caspase-2,GADD153, RE1α, -IRE1α, NK p-JNK Induce autophagy LC3I/II, Beclin-1	HeLa cells [62] HeLa cells [93]
Isoliquiritigenin From iquorice	Induce ROS Induce apoptosis Caspase-12	
3alpha,23-sopropylidenedioxyolean-12-en-27-oic acid (IPA)	Induce apoptosis Caspase-3,8,9 Mui-calpain GADD153	HeLa Cells [94]
Genistein from soybeans	Induce apoptosis Caspase-3 CHOP	HeLa cells [95]
Gambogic acid from Garcinia hanburyi	Induce apoptosis Caspas3,8,9, JNK	HeLa cells [96]

Protodioscin is a steroid compound derived from the rhizome of Chinese yam that has anticancer activity in a variety of tumors. It has been verified that endoplasmic reticulum stress is induced through activation of the MAPK signaling pathway, which is accompanied by high expression of GRP78 protein, thereby inducing apoptosis of cervical cancer HeLa cells. Mulberry yellow is a traditional medicinal mushroom. According to Shennong Materia Medica, “Mulberry Yellow” is used in medicine to solve the symptoms of gynecological tumors in the form of abdominal masses, including vaginal bleeding, leucorrhea, abdominal pain and abdominal masses. Studies have confirmed that the antitumor effect of Poria cocos extract on human cervical cancer SiHa cells occurs through inducing endoplasmic reticulum stress, which promotes high expression of GRP78 and CHOP, resulting in apoptosis induction in cervical cancer SiHa cells [97]. Tanshinone IIA is extracted from the dried roots of Salvia miltiorrhiza, a plant of the Lipopeae family, and it has a strong inhibitory effect on the growth of cervical cancer CaSki cells by promoting the caspase cascade and upregulating p38 and JNK signal phosphorylation. In addition to proteomics, network analysis, which shows overall protein changes, has indicated that Tan IIA causes apoptotic cell death by activating the ER stress pathway [98]. Studies have confirmed that the proliferation of HeLa cells is inhibited after treatment with 200–600 μg/mL fucoicin, which is extracted from plant kelp, for 48 h, while fucoicin treatment of normal cell lines, such as HaCaT keratinocytes and HEK-293 embryonic kidney cells, has no toxic effect, even though Bip/GRP78 and CHOP expression levels are increased. Thus, the accumulation of unfolded proteins in the ER is triggered, which induces apoptosis via the unfolded protein response (UPR) mechanism, resulting in cycle arrest of HeLa cells [99]. Moreover, studies have shown that compounds isolated from Sophora glycolipid species, which are obtained from plant matrix species by yeast, show toxic effects in HeLa and SiHa cervical cancer cells via inducing endoplasmic reticulum stress, upregulating GRP78 protein and promoting high expression of caspase 12 and caspase 3, ultimately inducing the apoptosis of cervical cancer cells. Furthermore, Sophora glycolipid species also inhibit the growth of xenograft tumors in nude mice in vivo [100].

## Conclusion and outlook

GRP78, as a molecular chaperone protein, is expressed in cervical cancer and affects the growth of tumor cells, the tumor microenvironment and tumor immunity. In response to changes in the external environment of cervical cancer cells, such as inflammation, hypoxia, nutrient deficiency and acidosis, GRP78 is overexpressed and migrates to the surface of tumor cells, thereby generating a stress protective response by supporting the promotion of tumor growth and inducing drug resistance of tumor cells and playing an important role in the growth, development and diagnosis of cervical cancer.

Apoptosis and autophagy determine the survival of tumor cells. In tumor cells, the interaction between autophagy and apoptosis is of great significance for the regulation of intracellular environment and clinical treatment. In cervical cancer, GRP78 plays a dual role of apoptosis and autophagy. On one hand, it plays an apoptotic role in cervical cancer cells, thus inhibiting the development of cervical cancer; on the other hand, it plays an autophagy role. GRP78 induces autophagy through endoplasmic reticulum induced UPR response, mild endoplasmic reticulum stress produces protective response, while sustained and strong endoplasmic reticulum stress induces a protective response. Malignant tumors constantly activate UPR under environmental stress, and ER response remains serious. GRP78 regulates tumor cell death signaling pathway and promotes tumor cell death. In recent years, further studies on the anti-tumor mechanism of endoplasmic reticulum stress also suggest that GRP78 plays an important role in the anti-tumor activity of natural plants, and GRP78 is involved in the synergistic effect of tumor immune response and HPV vaccine.

In terms of diagnostic significance, high expression of GRP78 is involved in the development of cervical cancer, and the differential expression of GRP78 in cervical cancer and adjacent tissues is correlated with the clinicopathological stage of cervical cancer patients and the differentiation of tumor cells, indicating that GRP78 is of great value as a diagnostic marker for cervical cancer.

How does GRP78 regulate cervical cancer in multiple intracellular sites? Does GRP78 regulate cervical cancer metastasis through EMT metastasis? It is necessary to establish GRP78 specific conditional knockout model and cervical cancer specific metastasis model, which will help to further reveal the function of GRP78 in cervical cancer and can be translated into the development of new cervical cancer treatment strategies in the future.

## Data Availability

Not applicable.
